# Gastric Cancer in Young Adults: A Different Clinical Entity from Carcinogenesis to Prognosis

**DOI:** 10.1155/2020/9512707

**Published:** 2020-03-02

**Authors:** Jian Li

**Affiliations:** Department of General Surgery, The Third Hospital of Mianyang Sichuan Mental Health Center, Mianyang, Sichuan 621000, China

## Abstract

Approximately 5.0% of gastric cancer (GC) patients are diagnosed before the age of 40 and are not candidates for screening programs in most countries and regions. The incidence of gastric cancer in young adults (GCYA) has declined over time in most countries except in the United States. Genetic alterations, environmental factors, and lifestyle may predispose some young adults to GC. According to molecular classifications, the cancer of most GCYA patients belongs to the genomically stable or microsatellite stable/epithelial-mesenchymal transition subtype, with the common genetic aberrations being mutations in *CDH1*. What characterizes GCYA are a higher prevalence in females, more aggressive tumor behaviors, diagnosis at advanced stages, fewer comorbidities and being better treatment candidates, and a similar or better survival outcome when compared with older patients. Considering the greater loss of life-years in younger patients, lowering the incidence of GC and diagnosing at a relatively early stage are the two most effective ways to decrease GC mortality. To achieve these goals, the low awareness of GCYA among general people, policy-makers, clinicians, and researchers should be changed.

## 1. Introduction

Gastric cancer (GC) remains an important cancer worldwide and it is estimated that there will be over 1,000,000 new cases and 783,000 deaths from GC in 2018, making it the fifth most frequently diagnosed cancer and the third leading cause of cancer death worldwide [1]. GC shows marked age variation and tends to be more frequently diagnosed in elderly patients with an average onset age of 68 years in the United States; more than 95% of all new cases are diagnosed in patients older than 40 years [2]. The incidence of GC has dramatically declined in recent decades; however, a stable or even slightly increasing trend in young adults has been reported [3]. Therefore, there has been an increasing interest in characterizing GC in young adults (GCYA).

Although young adults are less commonly affected by GC, previous reports have suggested that approximately 5.0% of GC patients are diagnosed before the age of 40 [2, 4, 5]. This is still a huge medical burden worldwide, especially for countries with a high incidence of GC. GCYA presents a challenge, in part because it is characterized by a high aggressive growth pattern and a more advanced stage at diagnosis, and many questions remain regarding carcinogenesis, treatment, prognosis, and prevention. Therefore, some authors have proposed that GCYA should be considered a different clinical entity, raising the necessity of differential management [2]. In this review article, I summarize the epidemiology, risk factors, molecular and clinical features, prognosis, and strategies for the prevention of GCYA, and provide some considerations for future perspectives.

## 2. Definition

The values used to define GCYA patients are not always consistent in the literature or guidelines. In some studies, early-onset GC is defined as GC before age 40, while in others, the definition generally includes all patients diagnosed before age 45. A younger or older age criterion has also been suggested, i.e., <35 or <50 years of age. Nevertheless, the majority of authors and large groups, including the National Cancer Institute and the Adolescent and Young Adult Oncology Progress Review Group (AYAO PRG), were in favor of the upper limit of 39 years of age in their studies [6, 7]. The incidence in females gradually changes from a higher to a lower level than that in males at 40 years of age, which represents a distinct feature of GCYA. In addition, survivorship studies across cancer types have similarly used age ≤40 to define young adults [8, 9]. For these reasons and for consistency and simplicity, we defined GCYA as tumors diagnosed before age 40. However, we must note that age is better appreciated as a continuous variable and variation exists in individuals of the same age; therefore, any predefined age cut-off is an arbitrary rather than an unequivocal definition.

## 3. Epidemiology

It is impossible to describe the epidemiological characteristics of GCYA exactly worldwide because of inconsistent completeness and accuracy of reporting across individual countries and regions. However, data generated by GLOBOCAN can be used to review secular trends and to make international comparisons.  Table 1 lists the incidence and mortality of GCYA in the top 10 countries sorted by the estimated number of incident cases in 2018 [10].

It is estimated that there were 26975 new cases of GCYA and 18063 associated deaths worldwide in 2018, ranking GCYA as the fifth most frequent cancer and the eighth leading cause of cancer in populations younger than 40 years. Asia, especially China, contributes to more than half of the incidence and mortality for the entire world, which is consistent with that for all ages. *Helicobacter pylori* (*H. pylori*) infection, environmental factors, and dietary components may partially explain the regional variation [1].

Global GC incidence and mortality rates have been declining over the last five decades worldwide, which may partially be due to the eradication of *H*. *pylori* infection, medical screening, and advances in the treatment [1]. However, the overall trend, which is an estimate by age-standardizing to the reference population, may mask the important age-specific features and geographic variability. Contrary secular trends of incidence rates of GC have been observed in young adults. In a study performed in the United States, the noncardiac GC incidence per 100,000 person-years among white adults aged 25 to 39 years increased from 0.27 in 1977-1981 to 0.45 in 2002-2006 [11]. In contrast, the trend for ages 20-39 expressed in annual percent change decreased at a rate of -3.7% and -0.8% in males and females, respectively, from 1999 to 2010 in South Korea [12].

**Figure 1:**
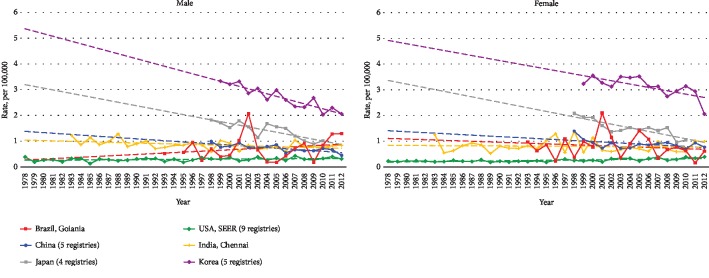
Trends in age-standardized incidence rate for young adults by sex; age < 40 years.

Whether in the East or West, almost all screening programs for GC are routinely carried out among middle-aged or elderly people [13, 14]; therefore, the differences in rates between countries and over time are likely to reflect real-life incidence rather than screening practices.  Figure 1 shows the secular trends of age-standardized incidence rates of GCYA in China, the United States, Japan, South Korea, India, and Brazil [10]. The overall age-standardized incidence rates of GCYA per 100,000 person-years declined over time for both sexes in Japan, South Korea, China, and India, and for females in Brazil, while for both sexes in the United States and for males in Brazil, an increased or flattened incidence rate has been observed in young adults.

## 4. Risk Factors

What characterizes cancer is a shared constellation of abnormal cell behaviors, such as rapid cell division and the invasion of surrounding tissue, which are linked to changes in DNA [15]. Cancer can affect anyone, while different genetic, environmental, and lifestyle factors may place some people at higher risk than others. Because of the extremely low incidence of GCYA, it is unrealistic to conduct large cohort studies to search for risk factors that act early in life. Therefore, all the studies are retrospective case-control studies with small sample sizes in the GCYA group. However, the same long-term trends of incidence rates between young adults and general-aged populations in some countries imply that they may share a number of the same risk factors. Also, the contrary long-term trends in some countries can help support that some factors may influence risk in young adults to a greater extent. In addition, characteristic clinicopathological features suggest that GCYA occurs under peculiar conditions.

### 4.1. H. pylori Infection

Soon after its discovery by Warren and Marshall, *H. pylori* was accepted as the main etiological factor in gastric carcinogenesis [16]. *The bacteria* can synthesize many different virulence factors to disrupt the balance between cell proliferation and apoptosis, which is an important driving force for the occurrence and development of GC [17]. Although *H. pylori* infection is considered to be a risk factor for the development of well-differentiated, intestinal-type GC in middle-aged or elderly populations, the etiological role of *H. pylori* infection in both diffuse-type and intestinal-type GCYA has also been elucidated. Pisanu et al. reported that GCYA patients had a significantly more frequent association with *H. pylori* infection after multivariate analysis [18]. The prevalence of *H. pylori* infection was reported to be higher in patients under 30 years of age with GC than in age- and sex-matched controls, and the positive rate in poorly differentiated adenocarcinoma cases was 95% [19]. Hirahashi et al. even found a significantly higher incidence of *H. pylori* infection in the young group than in the older group with intramucosal cancer of poorly differentiated type [20].

The familial clustering of GC may also partially be explained by *H. pylori* infection. Several studies have demonstrated that *H. pylori* infection clusters within families and may often be transmitted from parents to their children in early childhood as well as between siblings [21]. Studies have also reported that the prevalence of *H. pylori* infection and the incidence of precancerous lesions were high among the first-degree relatives of GC patients, and relatives of GC patients are more frequently colonized by the most virulent *H. pylori* cagA and vacA genotypes [22–24].

These observations strongly suggest that GCYA may be attributable to *H. pylori* infection. Along with the decline in the incidence of *H. pylori* infection resulting from screening and eradication programs, the incidence of *H. pylori*-associated GCYA has declined gradually in high-prevalence countries [25]. However, in the United States, a country with a generally lower prevalence of *H. pylori* infection, this trend has not been observed [26], which indicates that risk factors other than *H. pylori* infection may play a more important role in GCYA. In addition, *H. pylori* infection usually takes several decades to induce histological changes and subsequent neoplastic transformation [24], which suggests that different mechanisms underlie the carcinogenesis in younger and older populations, and further study on this aspect may be relevant to *H. pylori* infection to hereditary factor or immune-inflammatory response.

### 4.2. Hereditary Factors

Familial clustering was found in 10% of GC cases, and epidemiological studies have shown that the risk of GC in first-degree relatives is increased 2- to 3-fold [27]. This characteristic is more notable in GCYA. Studies from China, Korea, and Mexico all found that familial cancer aggregation is more common in GCYA patients than in older age groups [28–30]. The contributions of environmental effects such as *H. pylori* infection were discussed above; however, in GCYA, the contributions of inherited susceptibility may be more fundamental.

For GC, 1% to 3% of cases are a manifestation of several inherited cancer predisposition syndromes, including hereditary diffuse gastric cancer (HDGC), Lynch syndrome (also referred to as hereditary nonpolyposis colorectal cancer), juvenile polyposis syndrome (JPS), Peutz-Jeghers syndrome (PJS), familial adenomatous polyposis, and other less common hereditary cancer predisposition syndromes, which were discussed in detail in the NCCN guidelines of GC [31]. The vast majority of patients with GC affected by these syndromes are young. It was reported that the average age at diagnosis of HDGC is 37 years, and patients with Lynch syndrome have a 1% to 13% risk of developing GC, which occurs at an earlier age than in the general population [32, 33].

### 4.3. Hormones

Since the discovery of the presence of the estrogen receptor (ER) in some cases of GC, considerable controversy exists among studies on the relation between the ER and GC in the general population [34]. However, a higher female proportion is a near-universal finding demonstrated in each article for epidemiological studies of GCYA, which indicates that sex hormones, especially estrogen, may play an important role in GCYA development. In limited studies on hormone expression in GCYA, Zhou et al. showed that ER*β* rather than ER*α* expression is indeed correlated with young age and advanced cancer stages [35]. Matsuyama et al. reported that among signet ring carcinomas, ER*β* cytoplasm was stained in addition to nuclei, especially in GCYA [36]. A large case-control study also found that in females, frequent use of oral contraceptives without progesterone, older age at first delivery, a lack of lactation history, and nulliparity were significantly associated with an increased risk of GC [29], which may support the harmful role of estrogen in GCYA in females. A higher frequency of bone metastases in young patients may also be associated with estrogen receptor positivity, which has been demonstrated in other cancer types [2, 37].

### 4.4. Lifestyle

Other acquired risk factors, such as socioeconomic status, lifestyle, psychosocial environment, and dietary habits, are significantly associated with GCYA. Any use of tobacco products, weekly use of alcoholic beverages, higher intake of beef and barbecued/smoked foods, and lower intake of fresh fruits/vegetables are all associated with an increased risk of GC in young men, although their effects are present in different subsites [38]. A relationship between obesity and GC has not been identified definitely, although a hypothesis exists that increasing rates of gastroesophageal reflux disease (GERD) associated with obesity may predispose individuals to more proximal tumors. A number of studies have found a positive association between adolescent body mass index (BMI) and GC risk, either in overall GC or restricted to cardia GC [39]. However, few studies focus on GCYA, and further studies are needed, which may be very meaningful because the prevalence of overweight and obesity in childhood and young adults has been increasing worldwide in recent decades [40].

### 4.5. Industrial Materials

Currently, children and young people are more likely to be exposed to industrial materials than before, which may increase the incidence of many diseases, including cancer. Wu-Williams et al. reported that occupational exposure to metal dust was associated with a 70% increased risk of GC in young men [38]. Since decades ago, rapid industrialization has promoted the emergence of a large number of synthetic compounds, some of which can modify hormonal and homeostatic systems and thus interfere in the communication and response of an organism to its environment, classifying them as “endocrine disruptors” [41]. One study reported that the genes affected by “endocrine disruptors” were differently expressed in GCYA versus GC in old age. Therefore, the authors suggested that GCYA is the result of a genetic background that differs from that of GC in old age, and “endocrine disruptors” may play an important role in the carcinogenesis of GCYA [42]. However, this study is the only one available in the literature on this field, which needs to be explored further in the future.

## 5. Molecular Biology

GC is usually caused by cumulative genetic mutations and epigenetic alterations, but none of these are necessary or sufficient for cancer to occur; therefore, the molecular characteristics of GC are considered to be heterogeneous. Molecular analysis of data retrieved from The Cancer Genome Atlas (TCGA) has identified age-related expression changes in genes involved in the cell cycle, the muscle system process, and cell adhesion [43]. A key question about the molecular biology of GCYA is how genetic alterations can result in a malignancy in a relatively short period. This is far from definitely defined; however, our understanding of GC genetics was greatly expanded when two molecular classifications of GC were proposed by TCGA project in 2014 and the Asian Cancer Research Group (ACRG) in 2015. Most patients with GC belong to the genomically stable subtype in the TCGA classification or the microsatellite stable/epithelial-mesenchymal transition subtype in the ACRG classification are of young age, and the common genetic aberrations are mutations in *CDH1*, *RhoA*, or *CLDN18-ARHGAP* rearrangements; thus, it is no surprise that most of these tumors are characterized by higher rates of diffuse histological variants [44, 45].

### 5.1. CDH1

Germline truncating mutations in the *CDH1* gene are found in 30% to 50% of families with HDGC [46], and somatic mutations were detected in 42.2% of the young patients with diffuse-type GC, a value that was significantly higher than that of older age patients [47]. These findings might imply that *CDH1* mutations could be possible gene alterations that result in the early onset of diffuse-type GC in young patients. The protein encoded by the *CDH1* gene is E-cadherin, which is involved in the maintenance and homeostasis of the epithelium and can also transduce signals from the extracellular domain through the cytoplasmic tail into the nucleus to alter gene expression. The reduction or complete absence of E-cadherin is associated with a loss of epithelial morphology and increased invasiveness through epithelial-mesenchymal transition. The relation between E-cadherin and GC was reviewed by Liu and Chu in detail [48].

### 5.2. Ras Homolog Gene Family A (RhoA)

Acute cell death will occur when cellular adhesion is impaired by the loss of E-cadherin. For malignant transformation, other genes may play a synergistic role, such as *RhoA*, which encodes a small GTPase protein that plays a fundamental role in regulating diverse cellular processes [49]. In 2014, a TCGA study identified a rate of *RhoA* mutations and additional fusions in GTPase-activating proteins (GAPs), which are crucial in regulating RhoA activity. It also showed that genetic alterations in the *RhoA* pathway, along with the *CDH1* mutations, are quite common in diffuse-type GC but not in other variants of GC [44].

### 5.3. MSI

According to the ACRG analysis, microsatellite instability-high (MSI-H) tumors are enriched in elderly patients, while the microsatellite stable/epithelial-mesenchymal transition subtype presents at a significantly younger age with most of the patients diagnosed at advanced stages [45]. The results of other studies indicate that GCYA does not occur due to defects in the mismatch repair system [50].

### 5.4. Other Molecular Factors

In addition to these anomalies, Milne et al. summarized some other molecular profiles of GCYA, including the infrequent loss of TFF1 expression, low COX2 expression, no loss of RUNX3, and more frequent expression of low-molecular-weight isoforms of cyclin E [51]. Gene mutations associated with GCYA contributed to hereditary cancer predisposition syndromes: DNA mismatch repair (MMR) genes (*MLH1*, *MSH2*, *MSH6*, and *PMS2*) and the epithelial cell adhesion molecule (*EPCAM*) gene in Lynch syndrome, *SMAD4* or *BMPR1A* genes in juvenile polyposis syndrome (JPS), the *STK11* tumor suppressor gene in Peutz-Jeghers syndrome (PJS), and the adenomatous polyposis coli (*APC*) gene in familial adenomatous polyposis (FAP) [31].

Therefore, there are many possible genetic alterations that eventually initiate or promote the development of GCYA independently or in collaboration, and a clear-cut pattern of molecular characteristics does not exist. The current scientific challenge is to recognize which alterations play the most crucial role in particular patients and in particular stages to prevent the incidence of GCYA or to identify a treatment target.

## 6. Clinicopathogical Characteristics

An electronic search of PubMed was performed from January 2000 to October 2019 to identify studies that compared the clinicopathological characteristics of young and older patients with GC, and the age cut-off was limited to 40. A total of 19 studies were included [2, 4, 5, 52–67], from which we can draw the following characteristics of GCYA (Table 2):

### 6.1. A Higher Prevalence in Females

The sex distribution is a very distinctive feature of GCYA. However, some authors have concluded that the male-to-female ratio gradually changes from a female to a male predominance at 40 years of age. Most studies included in this review found a male predominance in GCYA, especially in studies that were based on national registration. However, compared with older patients, a higher prevalence in females was observed in GCYA in most studies, which was consistent with the incidence difference between different age groups. Before age 40, the incidence of GC was higher in the female population, while in those older than 40 years, the incidence of GC increased dramatically in the male population. The contributions to this difference are not clear, and potential explanations may involve two aspects. For women, hormonal changes may influence the incidence of GC, as discussed above. For men, they are more frequently exposed to known environmental risk factors, such as smoking and alcohol intake, which involves a sequence of preneoplastic lesions that take longer to develop, which contributes to increased GC incidence later in life.

### 6.2. More Aggressive Tumor Behaviors

Most studies revealed that higher grade, Borrmann type IV, signet ring cell, and diffuse-type cancers are more frequently diagnosed in young patients than in older patients. This disproportion may be primarily genetically determined, specifically alterations in the *CDH1* gene, which predispose individuals to GC at a young age with a diffuse phenotype. Diffuse-type GC lacks intercellular adhesion, which is often observed with diffuse invasion growth patterns throughout the stroma, characterized by rapid disease progression, being highly metastatic [68].

### 6.3. More Advanced Stage

Differences in TNM stage at diagnosis between young and older patients were found in most studies. Locally advanced and node or distant metastatic diseases are more frequently present in young patients. Except for highly aggressive growth patterns in young individuals, diagnosis delay may contribute greatly. A large proportion of GCYA may have no alarm symptoms, GC is not considered a differential diagnosis in young patients with gastrointestinal symptoms, and young populations are not assigned to endoscopic screening in various guidelines, which could delay investigation and diagnosis and result in a more advanced stage [69].

### 6.4. Fewer Comorbidities and Better Candidates for Treatment

There is no doubt that young patients with fewer comorbidities, especially those involving the cardiopulmonary systems, can tolerate more aggressive treatment. Data from the NCDB included 70084 patients and revealed that age may affect the treatment choice of doctors and patients. Young adults with stage I disease were more likely to receive chemotherapy or radiotherapy after the operation. For stage II and III disease, surgery+chemotherapy+radiotherapy is more often chosen for young adults. When stage IV disease is diagnosed, older patients are more prone to give up any treatment [2]. On the other hand, postoperative complications are closely related to comorbidities; therefore, a lower incidence of postoperative complications in young patients is beneficial to postoperative treatment [59].

## 7. Treatment

To date, therapeutic options for GC have not been stratified by age worldwide. According to the clinical guidelines of the Oncology Society, GCYA is not considered a criterion to drive treatment. In landmark trials on treatments for GC, patients included younger than 40 account for a very small proportion, and differences in response to treatment could not be inferred from these results. However, the clinicopathological and performance differences are important factors that determine the treatment choice of clinicians and patients.

### 7.1. Endoscopic Resection

For early gastric cancer (EGC), endoscopic resection, including endoscopic submucosal dissection (ESD) and endoscopic mucosal resection (EMR), has been an optimal modality in selected patients [70]. The indications for endoscopic resection are based on observational studies on the natural history of EGC in the general population [71]. Considering the more aggressive behavior in young patients with GC, the risk-benefit analysis of endoscopic resection should be finished before implementation. A study including 3741 patients with differentiated-type EGC showed that the lymph node metastasis rate in young patients was lower than that in older patients who fulfilled the endoscopic resection criteria, which validated the safety of endoscopic resection in these patients [72]. However, for undifferentiated EGC, which has been considered an expanded criterion for ESD in general patients, no literature is available to validate the safety of ESD in young patients. One study that included EGC containing undifferentiated-type histology cancers reported a higher lymph node metastasis rate in young patients than in older patients (38.3% vs. 13%) [64]. In addition, GCYA with a high proportion of diffuse-type tumors is partially associated with genetic alterations. Therefore, endoscopic resection for young patients with EGC with extended indications may not guarantee a good prognosis.

### 7.2. Surgery

Surgery is still the only chance for long-term survival for GCYA that can be curatively resected. Although Sun et al. reported that positive margins result in a significantly unfavorable outcomes for patients with relatively early-stage tumors but not for those with advanced diseases [73], some studies using multivariate analysis have indicated that status of resection margins, combined organ resection, and nodal involvement are independent prognostic predictors for GCYA [53, 74, 75]. These results may support an attitude that is worth verifying in future research that a more extensive surgery should be performed in young patients to achieve R0 resection and more lymph node harvest, with the advantage that these patients may be more likely to tolerate aggressive surgery. Alternative treatment strategies involving neoadjuvant chemotherapy or chemoradiation also need to be explored in young patients, although these strategies have proven to be effective in general-aged GC patients in pivotal clinical trials [76, 77].

### 7.3. Systemic Chemotherapy

In clinical practice, GC patients always present with unresectable advanced or recurrent disease, especially for young patients. For general-aged GC patients, the standard treatment regimen is systemic chemotherapy, based on the results of randomized controlled trials [78–80]. Although GCYA only accounts for a minority of cases in these RCTs, another study that was focused on young patients found that standard chemotherapy may have similar efficacy for these patients [81]. With favorable general conditions and organ function, GCYA patients experienced less adverse events, which may facilitate intensive therapy [81]. With regard to subsequent treatment outcomes after first-line chemotherapy, data were limited in GCYA patients. GCYA patients with diffuse-type GC tend to have peritoneal metastasis after first-line chemotherapy, and early detection is much more difficult than metastasis in other organs, which may worsen the outcomes [81].

### 7.4. Targeted and Immune Checkpoint Therapy

After publishing the ToGA trial, trastuzumab in combination with chemotherapy was considered to be a standard option for patients with HER2-positive advanced GC [82]. However, considering the histology of GCYA, the majority of tumors may be negative for HER2. According to the HER-EAGLE study, only 9.2% HER2 positivity was detected in patients before age 55 [83]. Based on the KEYNOTE trials, pembrolizumab shows promising antitumor activity in patients with heavily pretreated PD-L1-positive or MSI-H/dMMR advanced GC [84, 85]. Unfortunately, most GCYA patients belong to the genomically stable or microsatellite stable/epithelial-mesenchymal transition subtype in molecular classifications [44, 45]. Other checkpoint inhibitors have not shown any promising benefit in the treatment of GC [86, 87]. Therefore, patients with GCYA may not be good candidates for existing molecularly targeted agents and immune checkpoint inhibitors, which desire novel-targeted therapy developed by different approaches.

## 8. Survival

GCYA shares a more aggressive growth pattern, advanced tumor stage, and higher noncurability rate, which are all poor prognostic factors for GC affecting young and older patients similarly [67]. However, there are conflicting findings with respect to the survival of young patients. While some studies demonstrated poorer outcomes in young patients, the majority reported a better prognosis compared with older individuals, and some still have described no differences in survival based on age (Table 3). The controversy between studies can be explained by study limitations, era, and geographic variations. Variations among studies, including race, clinicopathological features, and treatment strategy, may contribute to inconsistent conclusions about survival. The stage distributions were different between young and older patients; however, patients in most studies were not matched based on tumor stage. Young patients with GC are most often diagnosed at advanced stage and organ involvement, suggesting a potentially greater burden of disease, which may lead to the poor long-term survival. In contrast, older patients, who are more often diagnosed early GC, may obtain better results. Therefore, when stage-specific survival was examined, young adults were demonstrated to perform as well as or better than older patients [2, 67]. This may be explained by the fact that young patients have fewer comorbidities or impairments of functional status and better tolerate aggressive treatment. In particular, treatment strategies have evolved over time. D2 surgery, minimally invasive surgery, perioperative management, and targeted therapy have been offered over the years and can significantly improve the prognosis in young patients, who are more often candidates for these treatments than their older counterparts [88]. In addition, when patients were classified into either young or all other ages, the survival was similar between the two groups, while when the middle-aged and elderly patients were grouped separately, the prognosis of the young and the older patients was significantly poorer than that of the middle-aged patients [54]. This reflects that the age categories chosen in studies are inherently arbitrary, and there may be differences in long-term survival for individual age subgroups.

Nevertheless, most studies lack information on cancer outcomes beyond OS, including disease-free survival, progression-free survival, metrics of response to treatment, or quality of life. In older patients, the main cause of death was comorbid conditions, while in young patients, it was due to advanced cancers. Therefore, the similar or better outcomes in GCYA patients are mainly due to youth itself, and this should not slow our search for ways to decrease the mortality of GCYA.

## 9. Prevention

Regardless of a similar or better survival outcome that can be obtained in GCYA patients, considering the greater loss of life-years in this younger population than in the general-aged population, we need strategies to decrease GC mortality in a young population. Before the emergence of a treatment that can cure any GC patient, lowering the incidence of GCYA and diagnosing at a relatively early stage are the two most effective ways to achieve that goal.

Unfortunately, lowering the incidence of GCYA by eliminating risk factors is unproven. Systemic review and meta-analysis showed that the eradication of *H. pylori* in asymptomatic, infected adults led to a reduced incidence of GC [89, 90]. Although the relationship between *H. pylori* infection and GCYA was not established exactly, a parallel incidence trend between *H. pylori* infection and GCYA and the benefits of *H. pylori* eradication persist for life all indicate that eradication in infected young populations is beneficial for preventing not only GC but also other related diseases, such as peptic ulcer, dyspepsia, and gastric mucosa-associated lymphoid tissue (MALT) lymphoma [91]. A similar concept also applies to other risk factors, such as lifestyle, dietary habits, and acquired environmental factors. The promotion of changes for some risk factors is probably unwise before carcinogenic or protective effects are elucidated, such as in the case of estrogen.

For hereditary factors, which are unchangeable with the current technological capabilities, endoscopic surveillance is recommended, even though safety and efficacy have not been established. For HDGC, available evidence suggests that endoscopy may not adequately detect precursor lesions. Therefore, prophylactic total gastrectomy (without D2 lymph node dissection) is recommended between the ages of 18 and 40 years for carriers of germline truncating *CDH1* mutations to prevent the occurrence of GC [31].

The most important prognostic factor for patients with GC is the tumor stage, and the long-term survival difference is significant among different stage tumors [92]. This indicates that early diagnosis in GCYA patients is beneficial for long-term survival. Mass screening programs that employ endoscopy have been introduced to detect early-stage GC in Korea and Japan, where GC is prevalent [93, 94]. However, the screening targets are limited to individuals 40 years or older because overuse of endoscopy is associated with a low yield rate in young patients and is not cost effective [95]. In addition, Park et al. reported that periodic endoscopies did not increase the proportion of young patients diagnosed with EGC [96]. These findings may hamper endoscopic screening for GC in young populations. However, there are several aspects worth our pondering. A study conducted by Park et al. included only patients with resectable GC, but it is still unclear whether periodic endoscopy can increase the discovery of resectable GCYA [96]. This may be the mainstay of the means to decrease the mortality of GCYA, in which EGC are difficult to detect by screening because of rapid progression. What is more, is it wise to calculate the cost-effectiveness by money when a loss of life-years is involved, especially in countries where the incidence of GCYA is high? Overall, young population-based screening is not achievable. However, the majority of GCYA patients presented with symptoms, most with dyspepsia, and minority with alarm symptoms [29]. A total of 17894 patients with simple dyspepsia underwent endoscopic examination, and 114 GC patients were diagnosed; 7 (6.14%) were younger than 40 [97]. In regions where the incidence of GCYA is higher, a large portion of cases would be missed if endoscopy was reserved for patients older than 40. Therefore, any patients with any stomach-related symptoms are candidates for upper endoscopic examination.

Another important difficulty is how to get young populations to avoid risk factors and participate in the screening campaign. Both in the Western and Eastern regions, the baseline awareness of GC risk factors and alarm symptoms is low, and the attitude towards GC screening is negative [98–100]. Therefore, acts with the goals of educating and increasing GC awareness among people including young nationally need to be carried out continuously.

## 10. Conclusions

**Figure 2:**
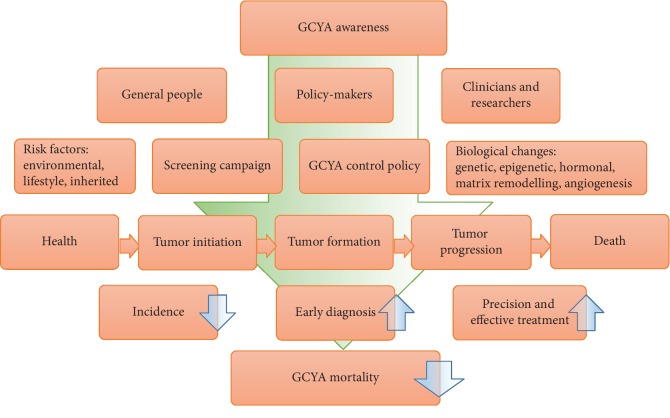
Efforts of general people, policy-makers, clinicians and researchers to decrease the mortality of GCYA. GCYA: gastric cancer in young adults.

GC is a growing threat in young adults, with many questions but few answers, and the threat has not always been taken seriously. What risk factors can result in a malignancy in such a short period are not known exactly, and even inherited predisposition may account for only a small proportion of cases. Although a similar or better survival outcome can be obtained in GCYA patients, this is mainly due to youth itself, with fewer comorbidities and better tolerance of aggressive treatment. With regard to the molecular mechanisms, precision treatment, and cost-effective screening methods, there are many questions awaiting our answers that require the efforts of all general people, policy-makers, clinicians, and researchers (Figure 2).

## Figures and Tables

**Table 1:** Incidence and mortality of gastric cancer to age 39.

Country	Incidence				Mortality			
	Numbers	ASR (world)	Crude rate	Cum. risk	Numbers	ASR (world)	Crude rate	Cum. risk
Worldwide	26975	0.46	0.55	0.02	18063	0.31	0.37	0.02
China	7236	0.66	0.97	0.03	4739	0.44	0.63	0.02
India	4360	0.40	0.46	0.02	3160	0.29	0.34	0.01
Brazil	969	0.56	0.74	0.03	518	0.29	0.40	0.02
Korea, republic of	939	2.4	4.0	0.13	187	0.48	0.79	0.03
Congo, Demographic of Republic	910	1.7	1.3	0.09	499	0.97	0.71	0.05
United States	681	0.30	0.40	0.02	316	0.14	0.18	0.01
Bangladesh	673	0.47	0.56	0.02	575	0.40	0.48	0.02
Vietnam	630	0.79	1.0	0.04	484	0.59	0.79	0.03
Japan	548	0.68	1.1	0.04	227	0.30	0.46	0.02
Russian Federation	548	0.48	0.74	0.02	428	0.34	0.54	0.02

**Table 2:** Clinicopathological differences between young and older patients with gastric cancer.

Variables	Higher in young patients	Similar	Higher in older patients	Features of GCYA
Female	[2, 4, 5, 52–54, 56–65, 67]	[55, 66]		Higher prevalence in females
Bormann IV	[5, 52, 58, 60, 62]	[4]		More aggressive tumor behaviors
Diffuse type	[4, 52, 53, 55, 57, 59, 64, 66, 67]	[62]		
Poorly differentiated	[2, 5, 52–63, 65]			
Stage IV	[2, 5, 57, 66]	[63, 67]		More advanced stage^a^
T4	[2, 5]	[67]	[55, 61]	
Lymph node metastasis	[2]	[5, 67]		
Distant metastasis	[2, 5]	[67]		
Comorbidity			[2, 4, 53, 59–61]	Fewer comorbidities and better candidates for treatment
No treatment			[2]	
Surgery	[2, 5, 57, 66]			
Adjuvant therapy	[2, 5, 57, 59, 66]	[53, 62]		
Postoperative complications		[4, 59]	[53, 57, 60]	

^a^GCYA: gastric cancer in young adults. Studies based on surgery data were excluded, because patients with advanced stages at diagnosis that are not candidates for surgery must have not been included in these studies.

**Table 3:** Prognosis of young patients with gastric cancer.

Study	Country	Period	Age groups (young/older)	No. of patients (young/older)	Survival (young/older)					
						All	Stage I	Stage II	Stage III	Stage IV
De et al. [2], 2018	USA	2004-2013	<40/≥40	2615/67469	5 y OS	21.1%/22.1%	65%/51%^∗^	45%/34%^∗^	30%/19%^∗^	5%/4%^∗^
Kulig et al. [4], 2008	Poland	1977-1998	≤40/>40	214/3217	mOS (resectable)	30.8 months/24.1 months				
					mOS (unresectable)	5.5 months/4.4 months				
Tavares et al. [55], 2013	Portugal	2000-2005	≤40/>40	23/360	5 y OS	47.6%/23.1%^∗^	83.3%/49.6%^∗^	62.7%/39.7%^∗^	0/8.53%^∗^	0/4.1%^∗^
Zheng et al. [56], 2013	China	2004-2006	≤40/>40	63/654	5 y OS	40.5%/55.6%^∗^				
Liu et al. [61], 2016	China	2008-2014	≤40/55-64	198/1096	5 y OS	62.8%/54.7%				
Isobe et al. [5], 2013	Japan	1977-2006	≤40/>40	169/3649	5 y OS	57.8%/64.3%^∗^	100%/97.3%	68.6%/76.7%	36.6%/37.4%	
					2 y OS					4.4%/10.4%
Hsieh et al. [53], 2012	China	1998-2006	≤40/56-75	115/1009	5 y OS	52.0%/53.0%	100%/92.3%	80.9%/66.4%	32.0%/32.2%	7.9%/2.9%^∗^
					5 y DSS	52.0%/50.3%				
Takatsu et al. [60], 2016	Japan	2000-2010	≤40/60-69	136/1435	5 y OS	80.6%/74.8%	*P* = 0.0631	*P* = 0.0439^a^	*P* = 0.189	*P* = 0.151
					5 y DSS	80.6%/79.5%	*P* = 0.481	*P* = 0.058	*P* = 0.468	*P* = 0.234
Kim et al. [59], 2014	South Korea	2002-2008	<40/≥40	112/112	5 y OS	84.3%/89.6%				
Saito et al. [54], 2012	Japan	1975-2000	<40/40-70/≥70	84/1314/587	5 y OS	61.0%/73.6%/68.1%^∗^				
Wang et al. [62], 2016	China	2005-2010	≤40/>40	342/3588	5 y OS	60.8%/53.7%^∗^	92.7%/92.5%	78.4%/70.2%	35.8%/28.8%	26.9%/10.3%
					5 y PFS	46.3%/40.5%^∗^				
Xu et al. [58], 2013	China	2001-2009	≤40/>40	99/894	5y OS	49.1%/44.4%				
Cormedi et al. [66], 2018	Brazil	2011-2013	≤40/41-65/≥66	71/129/94	2 y OS	31.0%/45.9%/35.1%	100%/80%/86.7%	56.2%/53.1%/65.5%^b^		3.1%/12.7%/5.3%^∗^
					2 y DFS	49.2%/59.2%/73.1%	80%/90.9%/90%	42.9%/47.5%/65.5%^b^		
Tekesin et al. [67], 2019	Turkey	1990-2014	≤40/>40	92/774	mOS	11 months/12 months				
Song et al. [65], 2017	China	2007-2011	≤40/>69	112/358	mOS	*P* < 0.001^a^	*P* = 0.207	*P* = 0.013^a^	*P* < 0.001^a^	*P* = 0.069

OS: overall survival; mOS: median overall survival; DSS: disease-specific survival; PFS: progression-free survival; DFS: disease-free survival. ^∗^The difference was statistically significant. ^a^The survival was better in young patients. ^b^Patients with stage II and III.
